# A Dermocosmetic Balm Containing Vitamin B5, Madecassoside, and a Prebiotic Complex Significantly Improves Post Fractionated CO_2_ Laser Downtime Versus a Repairing Skin Care: Results of a Randomized Double Blind Intra‐Individual Exploratory Study

**DOI:** 10.1111/jocd.16610

**Published:** 2024-10-03

**Authors:** Jean‐Michel Amici, Guénaelle Le Dantec, Ann' Laure Demessant, Catherine Queille‐Roussel, Magali Procacci Babled, Anne Claire Cathelineau, Alix Danoy, Solene Trevisan, Merete Haedersdal

**Affiliations:** ^1^ Service de Dermatologie Hôpital Saint André Bordeaux France; ^2^ La Roche‐Posay Laboratoire Dermatologique Levallois‐Perret France; ^3^ CPCAD CHU Nice Nice France; ^4^ Newtone Technologies–A QIMA Life Sciences Company Lyon France; ^5^ Department of Dermatology Copenhagen University Hospital Copenhagen Denmark; ^6^ Department of Clinical Medicine Faculty of Health and Medical Science University of Copenhagen Copenhagen Denmark

**Keywords:** dermocosmetic, post‐procedure, re‐epithelization, superficial CO_2_ laser, vitamin B5

To the Editor

Ablative fractional (Fx) CO_2_ skin resurfacing laser can reduce scars and alleviate aging signs by removing the superficial epidermal layers [1, 2]. However, temporary but bothersome side effects such as erythema, desquamation, and crusts may occur with a frequency and severity depending on laser settings which may impact the patient's quality of life (QoL) [2]. Therefore, experts recommend appropriate post CO_2_ laser skin care using dermocosmetics (DC) to restore the skin barrier integrity and to improve physical cutaneous signs [3, 4].

With this double‐blind, randomized intra‐individual study, we evaluated the skin re‐epithelization kinetics and barrier‐associated clinical signs and symptoms management with DC balm (Cicaplast® Baume B5+, La Roche‐Posay Laboratoire Dermatologique, France) containing vitamin B5, madecassoside, and a prebiotic complex including inactivated *Lactobacillus* spp. ferments, oligosaccharides, mannose, and *Aqua posae filiformis* *versus* a repairing DC (RDC) cream featuring thermal spring water, *Aquaphilus dolomiae* ferment filtrate, and purifying actives following Fx CO_2_ laser (LaserPulse®, Luminenis Ltd., France).

We included 25 adults, 15 women and 10 men, with a mean age of 37.7 ± 7.3 years and a Phototype II (3;12%) or III (22;88%). Clinical assessments comprised the assessment of the wound healing kinetic overtime based on re‐epithelization of the lesional area, the wound healing score (0 = none to 5 = complete healing), individual scores of erythema, desquamation and crusts (from 0 = none to 3 = severe) as well as their composite score (0–9). Moreover, the investigator assessed the skin color (ITA, individual typology angle) using a colorimeter (CL400 (E®), Courage & Khazaka, Germany) and took photos using SkinTone technologies (Newtone Technologies, France). The area under the curve (AUC) was calculated for the wound healing score and ITA values.

The mean complete healing kinetic was achieved significantly (*p* = 0.003) faster with DC balm (13.5 ± 3.1 days) than with RDC (15.8 ± 2.3 days); the mean LS difference was 2.771 days (CI 95%: 0.741; 4.801). The mean wound healing score (Figure 1) was significantly (*p* < 0.05) higher with DC balm compared to RDC between Day 6 and 10 and at Days 13 and 14 confirming a faster lesion healing with DC balm. At Day 17, 14 areas treated with DC balm compared to 10 treated with RDC were completely healed.

**FIGURE 1:**
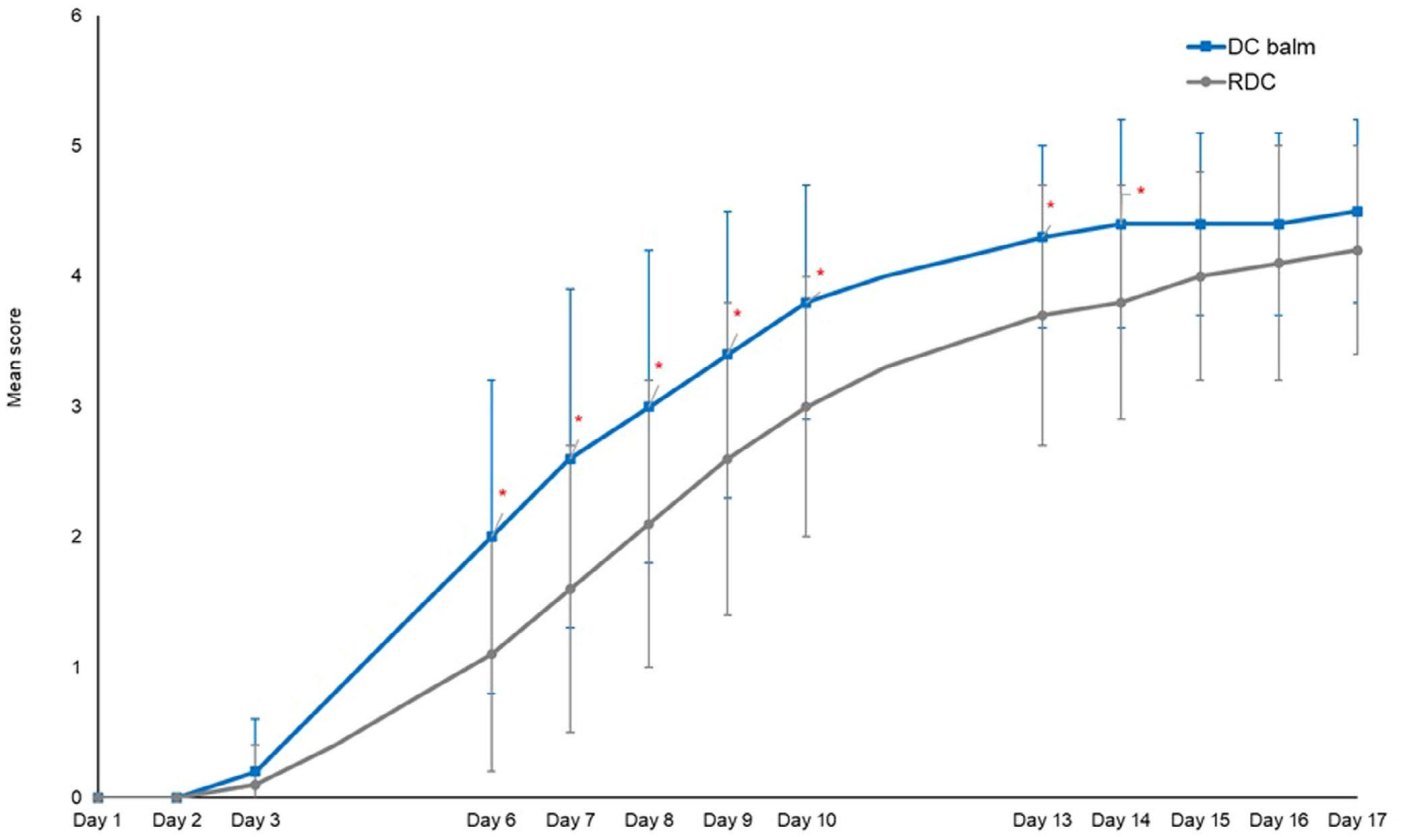
Evolution over time of the mean wound healing score. *p < 0.05 in favor of DC balm compared to RDC.

Furthermore, both the composite and crust scores had significantly (*p* < 0.05) faster and better improved compared to the RDC‐treated area between Day 6 and 10 as well as on Day 14. Nevertheless, we did not observe any significant differences between both products for the improvement of erythema and desquamation.

Finally, the AUC of the wound healing score was 44.9 ± 10.05 n/N (10.1%) for DC balm and 36.18 n/N (10.7%) for RDC. The difference was significantly (*p* < 0.01) in favor of DC balm (LS means: −8.72, CI 95%: −13.67; −3.74). The AUC for ITA was also significantly (*p* < 0.001; LS means: −136.79, CI 95%: −215.02; −58.56) in favor of DC balm 541.7 n/N (175.0%) over RDC 404.9 n/N (254.6%).

Figure 2 provides an example comparing the re‐epithelization of two zones treated with either DC balm or RDC.

**FIGURE 2:**
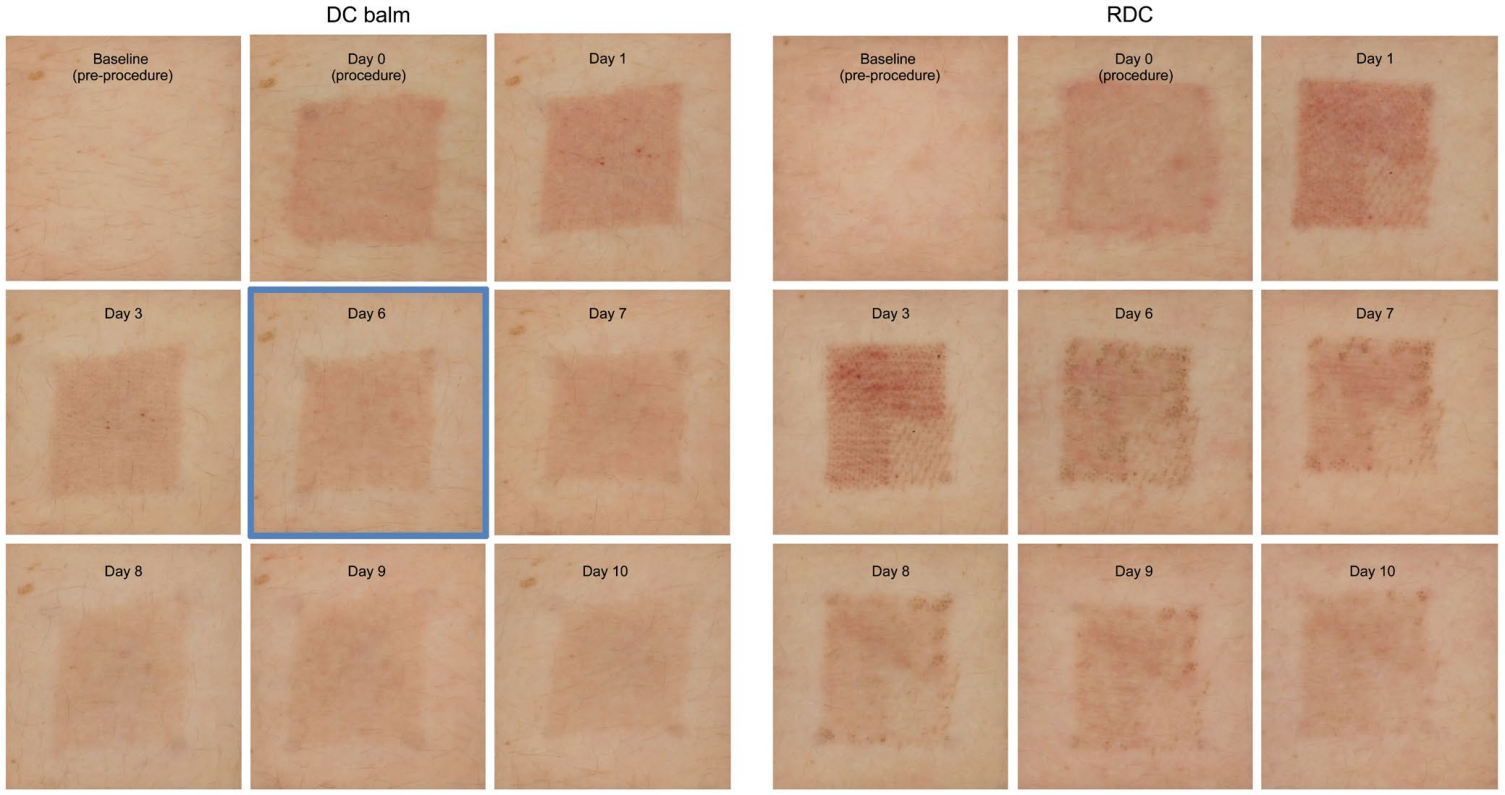
Examples of the re‐epithelization kinetics until complete wound healing of zones treated with DC balm (Day 6) or RDC (Day 13, not shown). Complete wound healing with DC balm was reached at Day 6.

In conclusion, this study demonstrated that a daily use of DC balm post‐Fx CO_2_ laser resulted in a significantly faster and better skin repair, especially during the first 14 days of application potentially improving the patients' QoL during this critical period. Moreover it significantly reduced crust formation and limited skin discoloration compared to RDC. The herewith presented exploratory results require confirmation through a large randomized study assessing not only the clinical benefits of the tested products but also the patients' quality of life status post‐laser.

## Authors Contribution

C.Q.R. performed the study, G.L.D. and A.L.D. supervised the study, all authors analyzed the data. G.L.D. and A.L.D. wrote, and all authors read and approved the manuscript.

## Ethics Statement

This single center, intra‐individual, randomized study adhered to the principles of Good Clinical Practices and the declaration of Helsinki. According to French regulatory guidelines, this type of trial received ethics committee approval (Comité Ethique Nord‐Ouest I on September 8, 2022, approval number 2022‐A01037‐36). All subjects provided written informed consent prior to their participation.

## Conflicts of Interest

Guénaelle Le Dantec and Ann'Laure Demessant are employees of La Roche‐Posay Laboratoire Dermatologique.

## Data Availability

The data that support the findings of this work are available from the corresponding author upon reasonable request.
